# Production System Monitoring Based on Petri Nets Enhanced with Multi-Source Information

**DOI:** 10.3390/s26061785

**Published:** 2026-03-12

**Authors:** Peng Liu, Xinze Li, Chenlong Zhang, Yanru Kang, Jun Qian, Weizheng Chen

**Affiliations:** 1 School of Mechanical and Aerospace Engineering, Jilin University, Changchun 130012, China; liupeng@jlu.edu.cn (P.L.); xzli9923@mails.jlu.edu.cn (X.L.); yanrukang_jlu@163.com (Y.K.); 2 Jilin Zhongwei Protective Equipment Co., Ltd., Changchun 130507, China; zcl2988@163.com; 3 School of Mechanical Engineering, Zhejiang University, Hangzhou 310058, China; 4 Engineering Training Center, Zhejiang University, Hangzhou 310058, China

**Keywords:** production line, condition monitoring, Petri nets, smart helmet

## Abstract

As the manufacturing industry continues to advance its digital transformation, intelligent sensing technology has become a key support for achieving precise, efficient and automated quality control. However, current production line monitoring systems predominantly rely on fixed and costly monitoring equipment and sensors, lacking flexible and interactive first-person perspective perception approaches centered on on-site operators. Meanwhile, factory process monitoring often depends solely on visual expression rather than balancing the capabilities of the simulation model and visual state detection, leading to delayed responses to abnormal systems and hindering the adjustment strategy feedback. To address these limitations, this study provides wearable sensing for key workers, enriching the state perception capabilities in industrial scenarios. Furthermore, to achieve dynamic model and real-time visual representation of production line operations, a multi-source information-enhanced Petri nets model is proposed in terms of engineering and user-friendliness. With the solid mathematical basics of the Petri nets and the enriched human–machine data from the product line, this method provides an intuitive, dynamic and accurate reflection of the production system’s real-time operational status, offering a scientific and reliable basis for operational decision-making. The proposed approach has been implemented in a real-world production system for reinforced concrete civil defense doors, and this engineering application can also be extended to many other scenarios.

## 1. Introduction

As the manufacturing industry increasingly shifts toward multi-variety, small-batch, and customized production models, mixed-model assembly lines have emerged as a critical enabler for discrete manufacturing enterprises to improve market responsiveness and optimize resource utilization efficiency [1]. Comprehensive system monitoring is indispensable for ensuring operational reliability, process stability, and performance traceability in assembly lines and manufacturing facilities. Conventional existing condition monitoring platforms lack awareness of human–machine status, simulation-based planning capabilities, and intuitive human–computer interaction, which collectively impede data-informed decision-making. Consequently, there is an urgent need to establish a comprehensive production monitoring system capable of capturing material flow, equipment status, and operator activities, while a real-time performance modeling evaluation method should be applied to enhance the analysis ability of monitoring [2,3,4,5].

Understanding production data and its flow is critical for system monitoring [6]. Nowadays, a human–machine monitoring approach is needed to enable comprehensive, real-time oversight of the entire production process [7,8,9]. In modern industrial settings, accurately identifying human–machine interaction states is key to automated monitoring and intelligent production management. Qadeer et al. achieved a trajectory prediction of mechanically thrown objects in modern industrial environments through multi-camera tracking and a Bi-LSTM deep neural network [10]. Research has made progress in recognizing human actions [11]. Yan et al. utilized a single 2D camera to estimate worker crowding density, achieving an error margin of approximately 0.6 m, albeit under the challenge of worker self-occlusion scenarios [12]. Wang et al. acknowledged the limitations of relying solely on single sensors for activity recognition in smart factories and thus adopted a multi-modal fusion approach by integrating IMU sensors with cameras to enhance accuracy [13]. Nonetheless, IMU sensors inherently possess drawbacks, including limited ranging distance and indirect position estimate, which result in relatively lower positional accuracy in industrial settings. Andronas et al. [14] combined visual, audio, and wearable sensor data to detect operator intent, boosting car assembly efficiency by 25%. EMG and video data together also improve detection of high-risk behaviors in dangerous tasks [15]. By combining multiple data sources, these methods expand the use of human–machine systems and support better behavior recognition in complex industrial environments.

After acquiring production system data, modeling methods are necessary for system analysis. There are three model categories: mathematical models (e.g., queuing theory, Markov chains), graphical models (e.g., flowcharts, Petri nets, GRAI nets), and simulation models (e.g., Plant Simulation, AnyLogic, FlexSim). Mathematical models require strong assumptions and perform poorly on systems with complex logic, high uncertainty, strong dynamics, or that are large-scale. Simulation models are tightly coupled to proprietary software, limiting reusability, portability, and modifiability. Graphical models, by contrast, offer both formal rigor and visual clarity, supporting system description, state monitoring, simulation setup and optimization design. Among them, Petri nets precisely model concurrency, synchronization, conflicts, and resource sharing, making them especially well-suited for dynamic behavior modeling [16]. For example, Cristian et al. [17] modeled the hydropower station’s in-service turbine governor using Petri nets. Mehdi et al. [18] embedded Petri nets into a network-based digital twin to model industrial IoT systems. Du et al. [19] proposed a Petri nets-based robust deadlock prevention method for automated manufacturing systems. Owing to the inherent flexibility of Petri nets modeling, numerous researchers have extended or adapted Petri nets to address domain-specific requirements. For example, Yang et al. [20] proposed a double-layer mapping Petri nets (DMPN) that separates information feedback and decision-making into two dedicated subnets—one for real-time monitoring, the other for closed-loop control—resolving the virtual–real mapping challenge and capturing event-driven system dynamics. Oliveira Júnior et al. [21] embedded Nelson’s rules and the Page–Hinkley algorithm into timed transitions, creating data-driven, AI-enhanced Petri nets for early detection of performance degradation and maintenance optimization in digital twins. Pla et al. [22] proposed Resource-Aware Petri Nets (RAPN), integrating time and resource constraints for intelligent workflows, enabling delay prediction and full-process monitoring in medical equipment maintenance. When integrated with anomaly recognition methods, Petri nets enable effective state monitoring and fault diagnosis in production systems. For example, Liu et al. [23] proposed self-modifying colored Petri nets (SMCPN) by combining colored Petri nets with the adaptive weight mechanism of self-modifying Petri nets, supporting systematic monitoring and diagnosis across multiple operational modes. Chen et al. [24] introduced the Petri net method for anomaly recognition in cigarette rolling, designed specifically to monitor air-tightness indicators with high sensitivity and interpretability.

Research shows that Petri nets and their extended forms, by integrating artificial intelligence, the Internet of Things, and fault tree analysis, can effectively describe the concurrent, asynchronous, and uncertain behaviors of complex systems, providing strong modeling support for system state monitoring, fault diagnosis, and performance optimization. However, for large-scale or complicated production systems, Petri nets models tend to suffer from combinatorial explosion in size and structural complexity, leading to diminished comprehensibility and limited effectiveness in visual representation and analysis. Existing methods still have significant shortcomings when modeling discrete manufacturing systems: material flow and process logic are ambiguously represented, making it hard to distinguish batch-specific paths. Resource usage and dynamic flows—especially for equipment and personnel under large-scale production—are poorly captured, limiting accurate load tracking. Task triggers like order initiation and process transitions lack intuitive expression, slowing on-site comprehension. Worker movements across processes and regions are not effectively modeled. Moreover, Petri net modeling is complex and inflexible, hindering real-time adaptation. These limitations impair clear visual monitoring and prevent practical use in real-time production control, restricting applications to theoretical contexts [25].

To address the above engineering issues, this study proposes multi-source information-enhanced Petri nets (MSI-Petri nets) for produce line monitoring. Section 2 analyzes the target production workflow and details the multi-source video fusion strategy, combining panoramic views, wearable camera feeds, and UWB positioning to capture operator behavior and equipment states. Section 3 introduces the method of MSI-Petri nets with mechanisms to improve visual expressiveness. Section 4 applies the approach in a civil defense door manufacturing setting. The main contributions are: (1) Real-time data and human–machine behaviors are integrated into the Petri nets for dynamic monitoring. (2) MSI-Petri nets place explicitly model humans, machines, and materials; material consumption ratios intuitively show inventory surplus. (3) Task-oriented transitions simplify the Petri net structure for diverse processes, and a time threshold visually indicates remaining transaction time, enabling engineers to monitor each product unit’s status at a glance.

## 2. Multi-Source Data Acquisition for Production Systems

### 2.1. Production System of Civil Defense Doors

Civil defense doors play an important role in urban infrastructure and in the disaster prevention and emergency response system. The product uses an integrated lattice structure, incorporating energy absorption, force mitigation, lightweighting, cost reduction, and ease of manufacturing and assembly into its design. This achieves both structural lightweighting and improved efficiency in production and assembly. This chapter presents a case analysis of the production process of civil defense doors at a manufacturing facility, focusing on data acquisition and operational status monitoring.

**Figure 1:**
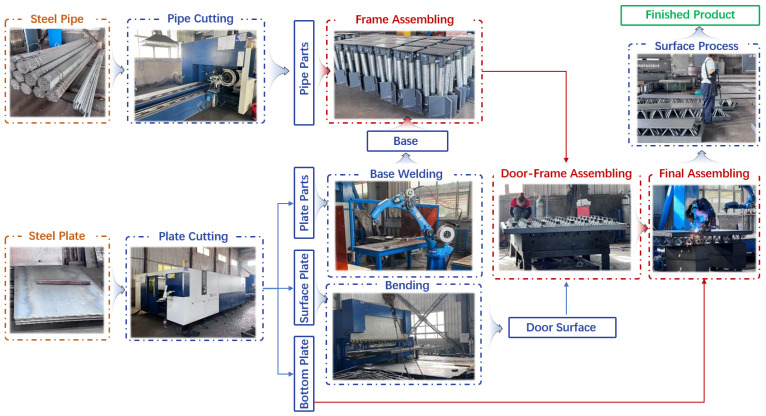
Process of the civil defense doors production system.

A comprehensive analysis of the factory’s layout indicates that the current production system is equipped with key machinery, including a pipe cutting machine, plate cutting machine, bending machine, and welding robots, with operators strategically assigned in accordance with the specific process requirements of each manufacturing stage. Given the present production scale, this configuration of equipment and human resources has established a stable and highly efficient process flow system, as illustrated in Figure 1. The raw materials for civil defense doors are steel pipes and steel plates. Steel pipes are cut to a specified length of approximately 30 cm using a pipe cutting machine, while steel plates are processed via a plate cutting machine into plate parts, surface plate, and bottom plate. Plate parts are welded into a base structure, which is then assembled with pipe parts to form the door frame. Surface plates are shaped into door surface components using a bending machine. These and other components are subsequently integrated to form a semi-finished product (door-frame structure). The bottom plate is then welded to the finished assembly to complete the structural integration. Finally, the assembly undergoes surface treatment processes, including deburring, rust removal, and painting, resulting in a finished product that complies with established factory specifications.

The status classifications of workers and machines were established based on eight core production processes of civil defense doors, namely, Pipe Cutting, Plate Cutting, Base Welding, Bending, Frame Assembling, Door-Frame Assembling, Final Assembling, and Surface Treatment, as detailed in Table 1. These states constitute the key state variables to be detected and identified by the data collection system proposed in this study. In Table 1, both human and machine states are uniformly represented using simplified coding: worker statuses are denoted by “HX” and equipment statuses by “MX”.

### 2.2. Design of Data Collection Method for the Produce System

This study presents a data acquisition system that integrates factory-wide monitoring with wearable devices for industrial workers. Helmets, as mandatory safety equipment continuously worn by personnel, offer inherent integration potential and consistent wearing stability, rendering them highly suitable as wearable sensing platforms. By embedding intelligent perception and feedback modules into helmets, the system enables efficient human–machine collaboration without imposing additional ergonomic load, while simultaneously capturing real-time production line status through the worker’s first-person perspective—yielding high-value, operation-critical data. To address scenarios where helmet usage is impractical, smart badges are introduced as alternative sensing terminals, preserving core data acquisition and interaction capabilities and thereby ensuring the system’s adaptability and operational flexibility across heterogeneous production environments.

In the domain of object recognition, YOLO (You Only Look Once) is a representative single-stage object detection framework that has been widely adopted across various computer vision applications owing to its high detection speed and accuracy. YOLOv8 further improves detection performance and inference efficiency through structural optimizations of the backbone network (CSPDarknet), the feature pyramid network (FPN), and the loss function. Nevertheless, conventional YOLO-based approaches exhibit limitations in modeling temporal dynamic information prevalent in industrial environments, particularly with regard to detection robustness under complex and cluttered background conditions. To overcome these challenges, this study proposes an enhanced YOLOv8 architecture integrated with multimodal temporal feature analysis, enabling more accurate and reliable recognition of dynamic interaction behaviors in complex industrial scenes.

**Figure 2:**
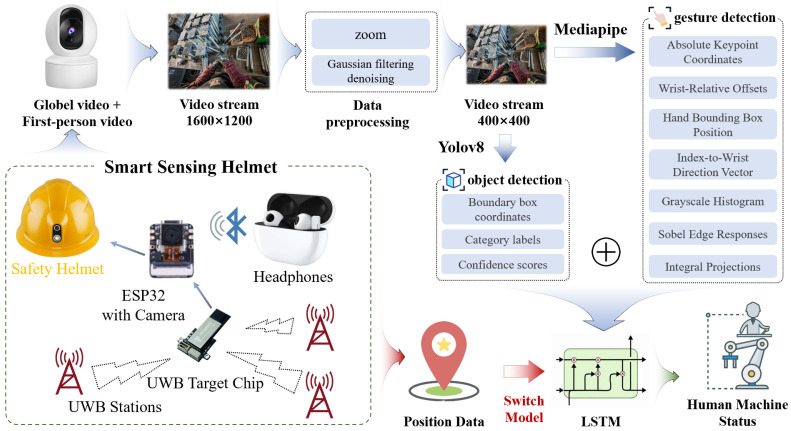
Schemes of data collection method.

The overall pipeline is illustrated in Figure 2. First, the system incorporates spatiotemporal localization data provided by Ultra-Wideband (UWB) devices to obtain the worker’s spatial position and dynamic motion state. Based on this information, the corresponding region- or task-specific recognition models are triggered, enabling on-demand activation and resource optimization.

Subsequently, a first-person video stream is captured by the camera mounted on the helmet, with an original resolution of 1600 × 1200 pixels. To achieve an optimal balance between computational efficiency and detection accuracy, all frames are uniformly resized to a fixed dimension of 400 × 400 pixels and subjected to Gaussian filtering to suppress image noise. On this basis, the YOLOv8 model is applied to detect key elements in each frame, identifying equipment components and materials. The detection results are aggregated into an 80-dimensional environmental context feature vector, which includes: the occurrence frequency of each object category, confidence scores, and normalized minimum/maximum bounding box coordinates along with center coordinates.

On the other hand, MediaPipe (0.10.21) is employed for hand gesture recognition, extracting 21 hand key points and constructing a 200-dimensional hand-perception feature vector that comprises:

- The original absolute pixel coordinates of each key point in the image coordinate system;
- Offset vectors of all key points relative to the wrist joint (i.e., key point #0);
- Position information of the hand’s minimum enclosing bounding box within the global image;
- A unit vector pointing from the tip of the index finger (keypoint #8) toward the wrist;
- Features extracted from the cropped hand region, including grayscale intensity histograms, horizontal and vertical edge responses computed via Sobel operators, and row/column-wise integral projections—collectively used to assist in inferring the worker’s actions and states.

These hand features and environmental context features are then concatenated into a 280-dimensional joint representation and organized as a time sequence, providing structured input for subsequent temporal modeling.

Finally, to address the need for in-depth temporal feature modeling, the algorithm employs ReID (Re-Identification) to perform cross-frame association of the same target across consecutive frames. By linking detected instances over time, it constructs the target’s motion trajectory and state transition sequence, thereby forming a comprehensive spatiotemporal state representation. To capture the long-term dependencies embedded within this sequence, an LSTM (Long Short-Term Memory network) is adopted to model and analyze the temporal dynamics. Leveraging its gated architecture, comprising the input gate, forget gate, and output gate, the LSTM effectively models long-range temporal correlations while mitigating the gradient vanishing problem inherent in conventional RNNs, thus enabling robust temporal understanding and classification of worker–device interaction states. Specifically, the model employs a two-layer LSTM architecture with an input dimension of 280 (combining 200-dimensional hand features and 80-dimensional object contextual features), a hidden size of 128, and a dropout rate of 0.3 to mitigate overfitting. During training, the Adam optimizer is used with an initial learning rate of 0.001, coupled with a StepLR scheduler (Learning Rate Scheduler) that halves the learning rate every 15 epochs; the model is trained for a total of 50 epochs with a batch size of 4. The model achieved a validation accuracy of 97.50%, with precision, recall, and F1-score all approaching 0.98. On a machine with a configuration of 9950X + 48G DDR5 + RTX 5090D, the model inference speed is approximately 8.2 ms. These results indicate that the proposed approach performs excellently in both real-time capability and accuracy. In cases where automated recognition remains ambiguous or uncertain, the system further validates the production process state through real-time interaction with the wearer via Bluetooth headphones, ensuring accurate state confirmation through human-in-the-loop feedback.

In conclusion, by leveraging multiple workers equipped with first-person sensing devices, a comprehensive factory-wide data-monitoring network is established to enable continuous acquisition of real-time time-series data pertaining to materials, equipment, personnel, and other operational elements. Edge computing is employed to perform data preprocessing and feature extraction at the source, thereby constructing multi-dimensional data models that support critical applications such as quality traceability, energy efficiency analysis, and process simulation. This architecture provides a robust data infrastructure for achieving manufacturing transparency and facilitating lean, data-driven decision-making.

## 3. Information-Enhanced Petri Nets

This chapter begins with a review of the classical Petri nets model for production cells, upon which a visualization mechanism is integrated to enhance its state representation capability. Subsequently, by incorporating the real-time data acquisition features of production lines and the dynamic attributes of actual production tasks, an information-enhanced Petri net theoretical framework is established, aiming to enable more accurate and intuitive operational monitoring of the production process.

### 3.1. Visualized Petri Nets for Produce Unit

A classical Petri net is a 4-tuple ***C*** = (***P*, *T*, *I*, *O***), where ***P*** denotes places: it is a finite non-empty set that characterizes resources, states, or buffer units in a production system; ***P*** = {*p*_1_, *p*_2_, …, *p_n_*}, where *n* represents the total number of places. *T* denotes transitions: it is a finite non-empty set that characterizes production operations or process nodes; it satisfies ***P*** *∩ **T** = Φ* and ***P*** ∪ ***T*** = Φ; $T=\{t_{1},\;t_{2},\ldots ,{\;t}_{m}\}$, where *m* represents the total number of transitions. ***I*** → 2*^P^* is the mapping from transitions to their input places; a single transition can be associated with more than one input place. ***O*** → 2*^P^* is the mapping from transitions to their output places; a single transition can be associated with more than one output place.

The marking of the Petri nets is represented by vector μ, where $\mu =\{\mu_{1},{\;\mu }_{2},\ldots ,{\;\mu }_{n}\}$ and $\mu_{i}$ denotes the number of tokens in the number *i* place. Therefore, marking can be used to characterize the transfer status of resources.

**Figure 3:**
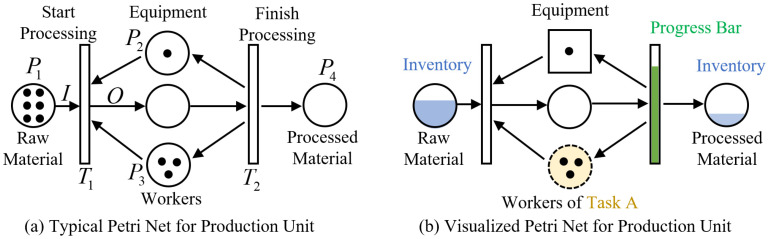
Petri Nets Model for production unit: (**a**) Typical Model, (**b**) Visualized Model.

A production system is composed of the organic integration of multiple production units, and a specific production link within the system can be modeled using the basic Petri net structure illustrated in Figure 3a. In this framework, material flow serves as the central thread, while various production requirements and process methods are represented by transitions. The places are: raw material (P_1_), equipment (P_2_), workers (P_3_), and processed material (P_4_); the places at the top denote the machine and equipment resources utilized, reflecting the occupancy status of these resources, whereas the places at the bottom represent the human resources assigned to the corresponding process. The transitions are: start processing (T_1_) and finish processing (T_2_). The marking of the Petri nets is μ={6,1,3,0}.

In condition monitoring, accurately capturing the real-time operational status is of critical importance. To this end, this study implements the following information enhancement strategies for the basic production unit, as illustrated in Figure 3b. First, given the wide variety and large quantity of raw materials, the filling degree is adopted in the visualization to represent the proportion of each raw material type relative to the total inventory volume, rather than directly displaying token counts, thereby improving readability and information density. Second, to effectively differentiate among the three types of places in the production system, namely, human, machine, and material resources, distinct visual representations are employed: worker places are depicted as circles with dashed borders, with those corresponding to workers capable of performing identical tasks filled with the same color; machine places are represented by solid-line rectangles; and material places are shown as solid-line circles. Third, the completion status of transition processes is dynamically displayed using a progress bar. A time factor is considered to quantify the time required for transition completion, while color variations in the progress bar are used to reflect the degree of deviation between the current state and the expected normal state.

### 3.2. Multi-Source Information-Enhanced Petri Nets

To address the requirements of production process modeling and monitoring for the entire civil defense doors production system, this paper proposes a multi-source information-enhanced Petri nets model (MSI-Petri nets). The core of this model lies in extending the elements and rules of traditional Petri nets, combined with further simplification and enhanced visualization, to better accommodate the key characteristics of complex production systems, such as resource state management, time constraints, handling of process interruptions, and personnel scheduling.

**Definition** **1.** *Structure of MSI-Petri Nets.*

The MSI-Petri nets contain 7 tuples, namely, (*P*, *T*_task_, *F*, *M*, θ, Γ, Σ), where each element is specified as follows:

**Place set** *P*: It is subdivided into three types of sub-places, namely, material places P_A_, equipment places P_M_, and worker places P_H_. These three types have different shapes and are easy to distinguish.

The material place adopts a visualization form of filling degree, which is determined by the material consumption ratio (current material number divided by daily consumption volume). This design can directly reflect the utilization level of material inventory. The equipment place and the worker place retain the traditional Token to mark the resource occupation status.

**Task-oriented Transition set** *T*_task_: It characterizes the start and end events of processing tasks. When equipment can switch between multiple processes, a task-oriented approach simplifies the system structure and supports condition monitoring.

Task switching mechanism: A mapping T_task_ → K (where K denotes the task set) is defined to bind production tasks to task-oriented transitions. During task switching, the ratio of the number of input arcs to output arcs, processing time, and visualization color of transitions all change dynamically, satisfying the following relationships:

(1) $$\left\{\begin{array}{c} \rho (t_{task},k)=\frac{number\;of\;input\;arcs}{number\;of\;output\;arcs}(k\in K)\\ \begin{array}{l} \tau (t_{task},k)\in R^{+}(processing\;time\;for\;task\;k)\\ color(t_{task},k)\in C(visual\;color\;for\;task\;k) \end{array} \end{array}\right.$$

Progress bar-based state deviation visualization mechanism: The task time of transitions is bound to the color and filling degree of the progress bar. Based on the standard working hours/historical working hours, when the actual processing time deviates from the benchmark value beyond the threshold, the color of the progress bar changes to issue an early warning. For worker assembly tasks, the progress bar is divided into filling intervals to match different assembly stages.

**Flow Relation** *F***:** A set of directed arcs satisfying F ⊆ (P × T) ∪ (T × P), which characterizes the material/resource flow between places and transitions. The flow velocity of arcs is visualized by the dynamic flickering frequency of the lines; when production is suspended, the arcs turn into dashed lines. The ratio of the number of input arcs to output arcs is determined by the material input-output ratio of the specific process.

**Marking Function** *M***:** A mapping M:P → N that denotes the number of tokens in places. For material places, the conventional token dots are replaced by “inventory container + filling progress bar”; for worker places and equipment places, traditional tokens are still used to indicate the availability status of resources.

**Time Threshold Set** θ: It is used to quantify the time boundaries for task execution and condition monitoring, with its mapping relationship defined as θ: T_task_ → (R^+^, R^+^). That is, for each task-oriented transition t_task_ ∈ T_task_, a pair of time thresholds ($\theta_{low},{\;\theta }_{up}$) is assigned. Among them, $\theta_{low}$ is the minimum process time threshold, representing the shortest effective duration for task execution; if the actual processing time is lower than this threshold, the process execution is deemed insufficient, and an early warning is triggered. $\theta_{up}$ is the maximum process time threshold, whose value is based on standard working hours or historically optimal working hours and increased by a certain proportion; if the actual processing time exceeds this threshold, the process is judged abnormal, and the color of the progress bar triggers an overtime warning.

**Resource Constraint Function** Γ**:** A mapping Γ: T→2^P^ that specifies the set of resource places required for transition firing.

**Cycle Parameter** Σ**:** A mapping Σ: F→R^+^ that assigns flow cycles to directed arcs, representing the transmission time of materials/resources on the arcs.

**Definition** **2.** *Transition Firing Rules of MSI-Petri Nets.*

Combining the characteristics of Time Petri Nets and progress bar visualization, the transition firing rules of MSI-Petri Nets are as follows:

For an MSI-PN = (*P*, *T*_task_, *F*, *M*, θ, Γ, Σ), a transition *t* ∈ *T* satisfies the enabling condition (denoted as *M* →) if and only if:

(1) Resource constraint is satisfied: For all *p* ∈ Γ(*t*), *M*(*p*) ≥ 1 (all required resource places contain at least one token).
(2) Input places are ready: For all *p* ∈ ∘*t* (where ∘*t* is input places), *M*(*p*) ≥ 1 (raw materials or intermediate products are available).
(3) Cycle constraint is satisfied: The cycle parameter Σ(*f*) corresponding to the flow relation *F* conforms to production requirements (the cycle value of the directed arc meets process specifications).
(4) Progress deviation is within the allowable range: The deviation between the filling degree of the progress bar and the standard working hours does not trigger the early warning threshold.

When the transition *t* fires (denoted as *M* → *M*′), the new marking *M*′ is updated according to the following rule:

(2) $$M^{\prime }(p)=\left\{\begin{array}{c} M(p)-1,p\in \circ t\cup \Gamma \left(t\right)\\ \begin{array}{l} M(p)+1,p\in \;t\circ \;\\ M(p),\mathrm{Otherwise} \end{array} \end{array}\right.$$

where *t*° represents the set of output places of transition *t*.

In terms of condition monitoring, when the production system is interrupted, the corresponding directed arcs turn into dashed lines, and a countdown for the buffer support duration is initiated. The duration time ($t_{duration}$) is determined by the current material number in the buffer ($N_{buffer}$) and the unit time consumption of the downstream process ($T_{unit}$):

(3) $$t_{duration}=\frac{N_{buffer}}{T_{unit}}$$

Which reflects the maximum time that the system can maintain operation after interruption.

## 4. Case Study

This chapter presents a case study of a civil defense doors production system and constructs the corresponding MSI-Petri nets, providing an effective visualization framework for monitoring and analyzing the operational states of the production system.

All factory work areas are equipped with stable lighting infrastructure, ensuring consistent illumination conditions; consequently, the influence of lighting variations on the visual recognition system is negligible. However, electromagnetic interference may occur when workers operate within one meter of large mechanical equipment, leading to reduced positioning accuracy, system latency, and positional drift, with fluctuations reaching up to ±0.8 m. Given the substantial physical separation between distinct operational zones, these localized disturbances have not significantly compromised the overall performance of the recognition system. Video data is compressed using the JPEG-based image format and transmitted via the TCP protocol, resulting in an average transmission delay of approximately 500 milliseconds. The data is wirelessly streamed in real time to a dedicated workstation capable of immediate processing.

**Figure 4:**
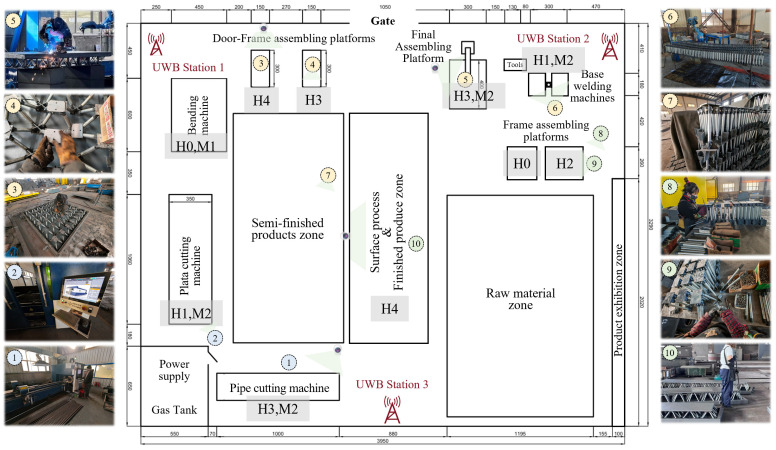
Human–machine status recognition of the production system.

The factory’s specific dimensions and spatial layout are illustrated in Figure 4. There are 10 workers on duty, categorized by job type: 2 male workers (labeled 1 and 2) are assigned to the preparation process (encompassing pipe cutting, sheet cutting, and sheet bending); 5 male workers (labeled 3 to 7) are dedicated to heavy tasks (welding, assembly, transportation); and 3 female workers (labeled 8 to 10) are responsible for light assembly and post-processing operations. This workforce allocation strategy takes into account both the physical demands and technical complexity of each process, supporting the intensive and rational utilization of human resources. Workers within the same category can be dynamically reassigned across different tasks and stations in response to real-time production scheduling, thereby enhancing operational flexibility and resource efficiency. Leveraging UWB positioning chips embedded in smart helmets worn by workers and 3 UWB base stations deployed throughout the facility, the system enables real-time, centimeter-level accurate tracking of each worker’s location, with their positions annotated numerically in Figure 4. Concurrently, visual data captured by helmet-mounted cameras or on-site cameras are displayed. By implementing the proposed multi-source perception algorithm, which integrates UWB-based localization with vision-based recognition, the system achieves precise, real-time awareness of “human-machine” states within each production unit, delivering reliable data support for dynamic process monitoring and optimization.

Based on the state classification coding scheme defined in Table 1 (where “HX” denotes human states and “MX” denotes equipment states), all relevant zones in the figure are annotated with corresponding state codes to facilitate intuitive interpretation. According to the recognition results: the pipe cutting unit is in a state of active operation involving personnel and equipment working (H3, M2); the plate cutting unit exhibits idle personnel with machine conducting plate parts cutting task (H1, M2); the base welding unit shows idle personnel while the equipment is engaged in welding (H1, M2); the bending unit has no personnel present but the equipment is working (H0, M1); the left and right frame assembly units are respectively unoccupied (H0) and undergoing fastening operations (H2); the left and right door frame assembly units are in states of worker fastening (H4) and worker calibration (H3), respectively; the final assembly unit involves collaborative welding between personnel and equipment (H3, M2); and in the surface treatment unit, the worker is performing painting operations (H4).

**Figure 5:**
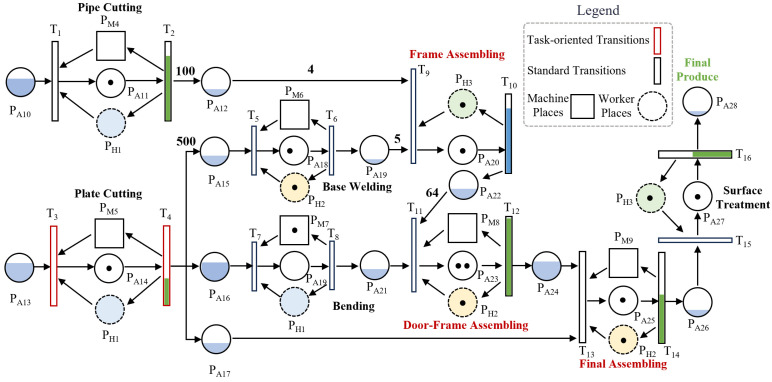
MSI-Petri net of the door production system.

As illustrated in Figure 5, the MSI-Petri nets model of the civil defense doors production system provides a comprehensive representation of system states. This model effectively captures the operational status of each production unit, particularly with respect to the utilization and flow dynamics of materials, human resources, and equipment resources. Transitions within the model serve as indicators of execution progress and enable the intuitive identification of abnormal conditions. The formal definitions of all places and transitions are provided in Table 2 and Table 3, respectively. By applying Petri net-based simulation and analysis methods, the dynamic behavior of the production system can be systematically modeled. Through techniques such as state reachability analysis and performance evaluation, the model facilitates the identification of production bottlenecks and supports optimized resource allocation, thereby offering a quantitative foundation for process improvement. Furthermore, the model enables traceability analysis of abnormal state propagation paths, aiding in the development of targeted exception-handling strategies.

In Figure 5, transitions T_3_ and T_4_ are designated as task-oriented, which means their output products vary depending on the specific task being executed, such as cutting plate parts, cutting surface plates, or bottom plates, and the processing time associated with each transition is accordingly task-dependent.

As illustrated in Figure 5, the Petri nets proposed in this study incorporate a progress bar-based state deviation visualization mechanism that dynamically links the task duration of each transition to the color and fill level of the corresponding progress bar. Taking T_2_, the transition associated with steel pipe cutting as an example, its reference task time Q1(T_2_) represents the predefined standard cutting duration. The fill height of the progress bar is positively correlated with the actual execution time, while its color changes adaptively based on the degree of temporal deviation: when the actual duration equals Q1(T_2_), the progress bar displays as “baseline green,” with the fill height precisely matching the expected duration; if the actual time exceeds Q1(T_2_) by 5%—for instance, due to increased processing time caused by equipment load fluctuations—the lower portion remains “baseline green” while the upper segment transitions to “warning yellow”; upon exceeding 10% or more, the majority of the progress bar shifts to “alarm red,” providing a clear visual indication of cumulative task-level deviation. This mechanism overcomes the inherent limitation of traditional Petri nets, which rely solely on token counts to represent system states, thereby enabling dual-dimensional visualization of both temporal progression and deviation magnitude. As a result, anomaly detection in production processes is advanced from post-event statistical analysis to real-time process monitoring, significantly enhancing the real-time monitoring and control capabilities of complex assembly systems.

The marking function has been enhanced with an inventory-oriented visualization extension, representing an innovative adaptation to large-scale material-handling environments. The fill ratio of the progress bar quantitatively reflects the current inventory utilization level. Furthermore, interactive access to individual places allows users to retrieve the exact quantity of stored materials upon clicking, enabling production operators to rapidly assess raw material availability and warehouse capacity utilization. This enhancement significantly improves information readability and operational control efficiency in complex, high-volume production settings.

Almost all production systems encompass fundamental elements, including: human, machines, materials, and methods, the same as Petri Nets for the Produce Unit. As such, by reorganizing and modifying the method developed in this study, it can be applicable to the modeling, monitoring, and analysis of more complex systems. By incorporating a task-oriented mechanism, the proposed approach can be effectively deployed in mixed-model production lines, enabling dynamic switching of operational rules across different stages.

In the current stage, the efforts were primarily focused on enhancing the readability of the production system model through human factors engineering, visual design, and simplification of information flow. The overall direction is recognized as one that can enhance user experience and efficiency. Following a period of practical deployment, operator feedback, such as data gathered via structured interviews and standardized questionnaires, can be conducted to further provide a comprehensive analysis of user-friendliness.

## 5. Conclusions

The primary engineering challenge in monitoring complex production lines is the absence of multi-source heterogeneous, real-time synchronized data capturing worker–device collaboration. To address this, we propose a unified perception framework integrating UWB positioning, YOLOv8-based object detection, and MediaPipe-based pose estimation, coupled with an LSTM model for temporal recognition of human–machine interaction behaviors. To overcome the lack of embedded simulation capability in conventional monitoring systems, we adopt Petri nets as the formal modeling backbone and enhance them with interactive, semantics-aware visualization—yielding an integrated system that simultaneously supports real-time status monitoring and dynamic process simulation. This system serves as a deployable engineering solution for refined operational oversight and closed-loop management of complex production lines.

Focusing on the manufacturing system of civil defense doors as the application scenario, a multi-source perception and visualization framework is proposed and implemented by integrating intelligent helmets with information-enhanced Petri nets. On-site operational data are collected through a network of factory global cameras and intelligent helmets, which, when combined with dynamic modeling via Petri net structures, enable real-time visualization and representation of production states. This integration significantly improves the transparency and controllability of the production process. Case study results demonstrate that the proposed method enables recognition and monitoring of human–machine interactions, thereby providing reliable data support for production decision-making. With the foundation of the proposed MSI-Petri nets, future research can advance toward simulating the production system, enabling optimization decisions, and evaluating model performance based on real-world operational efficiency at the site. Furthermore, the method functions as a structured representation of a digital twin, facilitating not only real-time monitoring but also system-level analysis and process control, thereby offering robust decision-making support for expert systems.

The MSI-Petri nets model incorporates multiple information sources, including normalized resource identifiers, temporal attributes, arc state constraints, personnel scheduling logic, and equipment selection transitions, effectively overcoming the limited observability and interpretability inherent in traditional Petri nets within complex manufacturing environments. As a result, it facilitates fine-grained and visually interpretable modeling of production workflows. While this research successfully addresses monitoring requirements in a specific industrial context, its underlying technical framework exhibits strong potential for generalization to other manufacturing domains, offering practical insights for advancing industrial intelligence. Future work will focus on adaptive model optimization, cross-scenario generalization, and the application of the MSI-Petri nets to multi-objective optimization in production systems.

## Figures and Tables

**Table 1:** Classification of the status of different production units.

Production Unit	Status Classification
Pipe Cutting	**Human:** H0 (None), H1 (Idle), H2 (Loading materials), H3 (Operating), H4 (Unloading materials)
	**Machine:** M1 (Idle), M2 (Working)
Plate Cutting	**Human:** H0 (None), H1 (Idle), H2 (Loading materials), H3 (Operating), H4 (Unloading materials)
	**Machine:** M1 (Ideal), M2 (Plate Parts Cutting), M3 (Surface Plate Cutting), M4 (Bottom Plate)
Base Welding	**Human:** H0 (None), H1 (Idle), H2 (Loading materials), H3 (Operating), H4 (Unloading materials)
	**Machine:** M1 (Idle), M2 (Working)
Bending	**Human:** H0 (None), H1 (Idle), H2 (Loading materials), H3 (Operating), H4 (Unloading materials)
	**Machine:** M1 (Idle), M2 (Working)
Frame Assembling	**Human:** H0 (None), H1 (Idle), H2 (Install support), H3 (Fasten), H4 (Transporting)
Door-Frame Assembling	**Human:** H0 (None), H1 (Idle), H2 (Replace), H3 (Calibration), H4 (Fasten), H5 (Welding), H6 (Transporting)
Final Assembling	**Human:** H0 (None), H1 (Idle), H2 (Replace), H3 (Welding), H4 (Transporting)
	**Machine:** M1 (Idle), M2 (Welding), M3 (Transporting)
Surface Treatment	**Human:** H0 (None), H1 (Idle), H2 (Deburring), H3 (Dedusting), H4 (Painting)

**Table 2:** Definition of places in Petri nets.

Place	Name	Place	Name
P_H1_	Workers of cutting, bending tasks	P_H2_	Workers of welding task
P_H3_	Workers of assembling task	P_M4_	Pipe cutting machine available
P_M5_	Plate cutting machine available	P_M6_	Welding machine available
P_M7_	Bending machine available	P_M8_	Assembling platforms available
P_M9_	Final assembling machine available	P_A10_	Pipe raw material
P_A11_	Pipe cutting in process	P_A12_	Pipe parts
P_A13_	Plate raw material	P_A14_	Plate cutting in process
P_A15_	Plate parts	P_A16_	Surface plate
P_A17_	Bottom plate	P_A18_	Welding in process
P_A19_	Base	P_A20_	Bending in process
P_A21_	Door surface	P_A22_	Frame
P_A23_	Door-Frame assembling in process	P_A24_	Door-Frame
P_A25_	Final assembling in process	P_A26_	Complete door
P_A27_	Surface treatment is in progress	P_A28_	Final product

**Table 3:** Definition of transitions (T) in Petri nets.

T	Name	T	Name
T_1_	Pipe cutting begins	T_2_	Pipe cutting finished
T_3_	Plate cutting begins	T_4_	Plate cutting finished
T_5_	Base welding begins	T_6_	Base welding finished
T_7_	Bending begins	T_8_	Bending finished
T_9_	Frame assembling begins	T_10_	Frame assembling finished
T_11_	Door-Frame assembling begins	T_12_	Door-Frame assembling finished
T_13_	Final assembling begins	T_14_	Final assembling finished
T_15_	Surface treatment begins	T_16_	Surface treatment finished

## Data Availability

The data presented in this study are available on request from the corresponding author.
